# Inefficiency of public hospitals: a multistage data envelopment analysis in an Italian region

**DOI:** 10.1186/s12913-021-07276-5

**Published:** 2021-11-27

**Authors:** Luca Piubello Orsini, Chiara Leardini, Silvia Vernizzi, Bettina Campedelli

**Affiliations:** Department of Business Administration, University of Verona, Via Cantarane 24, 37129 Verona, Italy

**Keywords:** Data envelopment analysis, Efficiency, Quality, Public hospitals, Tobit, Malmquist productivity index

## Abstract

**Background:**

The objective of this study was to assess public hospital efficiency, including quality outputs, inefficiency determinants, and changes to efficiency over time, in an Italian region. To achieve this aim, the study used secondary data from the Veneto region for the years 2018 and 2019.

**Methods:**

A nonparametric approach—that is, multistage data envelopment analysis (DEA)—was applied to a sample of 43 hospitals. We identified three categories of input: capital investments (Beds), labor (FTE), operating expenses. We selected five efficiency outputs (outpatient visits, inpatients, outpatient visit revenue, inpatient revenue, bed occupancy rate) and two quality outputs (mortality rate and inappropriate admission rate). Efficiency scores were estimated and decomposed into two components. Slack analysis was then conducted. Further, DEA efficiency scores were regressed on internal and external variables using a Tobit model. Finally, the Malmquist Productivity Index was applied.

**Results:**

On average, the hospitals in the Veneto region operated at more than 95% efficiency. Technical and scale inefficiencies often occurred jointly, with 77% of inefficient hospitals needing a downsizing strategy to gain efficiency. The inputs identified as needing significant reductions were full-time employee (FTE) administrative staff and technicians. The size of the hospital in relation to the size of the population served and the length of patient stay were important factors for the efficiency score. The major cause of decreased efficiency over time was technical change (0.908) rather than efficiency change (0.974).

**Conclusions:**

The study reveals improvements that should be made from both the policy and managerial perspectives. Hospital size is an important feature of inefficiency. On average, the results show that it is advisable for hospitals to reorganize nonmedical staff to enhance efficiency. Further, increasing technology investment could enable higher efficiency levels.

## Background

The recent global economic crisis has influenced the budgets of public organizations, including those of public hospitals and national health systems in general. According to the World Health Organization, for high-income and upper-middle-income countries, such as Italy, the main challenge relating to the provision of health services is to continue improving efficiency, quality, and equity [1]. Moreover, within the evolving social and economic environment, budget constraints have prompted a search for new ways of monitoring and controlling organizational finances that focus on the efficient and effective use of public resources [2]. Monitoring the performance of healthcare providers is a relevant issue worldwide, particularly in contexts such as hospitals given their significant effect on population health and the economy. The main problem facing hospitals has been inefficient use of existing resources rather than a lack of resources [3]. Therefore, hospital efficiency plays a strategic role in healthcare organizations. Assessing the determinants of efficiency allows managers to formulate appropriate organizational strategies to meet the challenges associated with continuous change. Focusing on the Italian National Health System, Guerrini et al. [4] note that in recent years, increasing attention has been paid to ensuring financial equilibrium and reducing the average annual growth rate of total health expenditure per capita. Italy has a regionally based National Health System that provides universal coverage free of charge [5]. Regional governments allocate resources to healthcare organizations and have a significant degree of autonomy in organizing the provision of healthcare planning and monitoring, as well as determining the number and vocation of healthcare providers [6], and the size of hospitals in terms of number of beds. One of the most important choices of Italy’s regional governments in relation to hospitals is the number of beds.

### Literature review

Over the past 30 years, many research studies on hospital efficiency evaluation have been conducted in different countries. Färe et al. [7] published the first European study, and this was followed by many others [8–13]. Such studies have now been conducted in many other countries around the world, including in China, Iran, Brazil, Ukraine, and Angola [14–18].

Hospital efficiency has been evaluated in relation to many factors (see Table 1). Kohl et al. [19] consider capital investment of great importance in efficiency analysis. Chilingerian and Sherman [20] note the importance of labor to the service process in hospitals, identifying this factor as essential in assessing performance. In addition, other organizational choices, such as teaching status and the provision of first aid, are often considered relevant in assessing hospital efficiency in the literature [21]. A frequently investigated aspect of hospital efficiency is patient length of stay. Among previous studies there is consensus that an increase in the number of hospitalization days has a negative effect on hospital performance [22, 23]. Rebba and Rizzi [24] have found that a high number of beds per inhabitant is one of the major causes of hospital inefficiency because it increases overhead costs. In addition, Daidone and D’Amico [25] and Shahhoseini et al. [26] argue that efficiency is positively affected by hospital size. Nevertheless, this relationship remains controversial because research such as that of Nayar et al. [27] has found that small hospitals have higher efficiency and quality scores than do large hospitals. In addition, Chang [28] argues that the number of service types offered is negatively related to efficiency because a greater scope of service means a higher level of management complexity. This is supported by Campedelli et al. [29], who found that hospitals that provide a first-aid service incur higher costs than hospitals that do not provide this service.

**Table 1:** Factors affecting hospital efficiency

Factors affecting hospital efficiency	References
Capital investment	Kohl et al. (2019) [19]
Labor	Chilingerian and Sherman (2011) [20]
Teaching status	Kakeman et al. (2016) [21]
First aid	
Length of stay	Staat (2006) [22]; Dimas et al. (2012) [23]
Size	Rebba and Rizzi (2000) [24]; Daidone and D’Amico (2009) [25]; Shahhosein et al. (2011) [26]; Nayar and Ozcan (2013) [27]
Number of service types offered	Chang (1998) [28]; Campedelli et al. (2014) [29]

Many studies on hospital efficiency assess pure technical efficiency (PTE) and scale efficiency [30, 31]. PTE represents managerial efficiency [32], which refers to management’s ability to save inputs to produce a certain amount of outputs or to produce more outputs with a given level of inputs [33, 34]. Scale efficiency indicates whether an organization operates at the most productive scale size [35].

However, despite the numerous studies on hospital efficiency conducted in many different countries, few studies have attempted to include quality measures [36], particularly those undertaken in the European context. One possible reason for this gap could be that the scientific community has not yet agreed on a common standard for addressing questions of quality in hospitals [19]. However, according to Chatfield [37], efficiency studies without quality considerations are neglecting a critical factor. Some research that does consider quality has been conducted in the United States [38, 39], and it identifies mortality rate as a good valuation of quality.

The purpose of this study was to assess the efficiency of public hospitals while including measures of quality. To fulfill this aim, the study attempted to answer the following research questions: (1) What are the main organizational factors that generate hospital inefficiency? (2) How do internal and external features affect hospital efficiency? (3) How has hospital efficiency changed over time? To answer these questions, the study employed multistage data envelopment analysis (DEA).

### Data envelopment analysis

DEA is a nonparametric technique developed by Charnes et al. [40]. It is used to rank and compare the efficiency of various entities, defined as decision-making units (DMUs). DEA is grounded in an optimization algorithm that assigns a score between 0 and 1 to the DMUs given the input consumed and the output produced. DEA models allow assessment of the relative efficiency of DMUs by creating a production frontier using the best practice of the observed data. In addition, DEA can be considered an alternative to parametric frontier methods and financial ratio analysis. Rhodes [41] highlights that financial ratios allow benchmarking among a multitude of operating units, focusing on their financial results. Nayar et al. [27] note that the main flaw in measuring performance using this kind of methodology is the lack of technical indicators that enable evaluation of the efficiency of structures and the quality of services provided. According to Worthington [42] and O’Neill et al. [43], methods of nonparametric analysis such as DEA overcome the weaknesses of financial ratios and parametric analysis because they do not require any assumption related to the functional form of the relationship between outputs and inputs [44]. Further, DEA can not only identify inefficient units but can also assess the degree of inefficiency. DEA uses linear programming to construct a piecewise convex linear-segmented efficiency frontier, making it more flexible than econometric frontier analysis. Moreover, DEA can include multiple inputs and outputs. Despite these identified benefits, DEA presents a drawback: it attributes every deviation from the best practice frontier to inefficiency. However, such deviations might be due to statistical noise (e.g., measurement errors).

## Methods

### Study design

The study adopted a cross-sectional design to assess the efficiency of public hospitals in the Veneto region for the years 2018 and 2019. It used a longitudinal design to analyze the trend in technical efficiency in general hospitals from 2018 to 2019. More specifically, a Charnes, Cooper, and Rhodes (CCR) input-oriented model, decomposition of the obtained scores, and slack assessment were developed for 2018 and 2019. Subsequently, Tobit regression was applied to understand the internal and external sources of inefficiency. Finally, the Malmquist Productivity Index was used to assess how efficiency has changed over time.

### Study population

To conduct the efficiency analysis, the Veneto region was selected as the case study site [45]. This was because of the region’s high level of interest in researching new ways to control the efficiency of public hospitals. Guerrini et al. [3] found that this region has specific characteristics (i.e., an increase in the elderly population and growing life expectancy of residents) that have led to a gradual increase in comorbidity and chronic diseases and a corresponding increase in demand for high-quality healthcare services. All the data used in this study were provided by Azienda Zero UOC Controllo di Gestione e Adempimenti LEA (Azienda Zero). A full dataset from 2018 to 2019 containing nonpublicly available technical data cost and revenue items was analyzed. The data included in this study are the operative costs and revenue for public hospitals, number of beds, number of FTEs, mortality rate, inappropriate admission rate, bed occupancy rate, length of stay, provision of first aid, and number of residents. During the period under analysis, the Veneto region had 53 public hospitals. One hospital closed in 2018, three hospitals treated only a particular type of patient and were therefore considered nonhomogeneous and noncomparable, and six hospitals presented missing data. Therefore, the final sample included 43 hospitals and 86 observations.

### Selection of study variables

Selecting suitable inputs and outputs is crucial for ensuring meaningful efficiency analysis. To obtain the discriminative power of DEA, Dyson et al. [46] recommend being parsimonious with the number of inputs and outputs. According to these researchers, the number of DMUs should always be larger than two∙(#inputs + #outputs) to ensure sufficient discrimination between units. In this research, six inputs and seven outputs were chosen. Therefore, this rule was not violated.

Following Ozcan [47], we used three categories of inputs: capital investment, labor, and operating expenses. As suggested by Kohl et al. [19], a good proxy for capital investment is the number of beds (Beds). This variable is widely used in the literature [48–51]. As a proxy for labor, we used the number of hospital FTEs. Chilingerian and Sherman [20] note the relevance of labor to hospital efficiency. In addition, these researchers advise distinguishing between different types of personnel. Thus, we identified four categories of personnel: medical (FTE Med), nursing (FTE Nurse), administrative (FTE Admin), and technical (FTE Tech). As a proxy for operating expenses, we used the operating costs (Cost) of hospitals, which is a commonly used variable in the literature [52–54].

For the outputs, we used five variables to evaluate hospital efficiency and two variables to evaluate hospital quality. For efficiency-output variables for hospitals, Ozcan [47] advises including inpatient and outpatient visits. Thus, we used the number of outpatient visits (Outpatients) and the number of case-mix-adjusted inpatients (Adj. Inpatients). A commonly used output is operating revenue [55–57]. To measure this output, we used revenues from inpatient visits (Inpatient Revenue) and revenues from outpatient visits (Outpatient Revenue). Another variable commonly used to analyze hospital efficiency [58, 59] is bed occupancy rate (BOR), which we also included as an efficiency-output measure. We used two measures to evaluate hospital quality as an output. The first quality measure was mortality rate, which is often used in hospital efficiency analyses that incorporate quality [39, 60, 61]. The second quality measure is a new variable that has not been used in previous literature: the potential inappropriate admission rate, which is the ratio between admissions attributed to diagnosis-related groups at a high risk of inappropriateness and admissions attributed to no risk of inappropriateness. This measure represents the hospital’s ability to classify patients and receive adequate remuneration for hospital activities. These two quality variables are considered undesirable outputs. Incorporating undesirable outputs in DEA research has been discussed in previous studies. One idea [62] is to apply inversion transformation to the value of undesirable factors. However, the transformed values are usually negative, making it difficult for DEA modeling. To overcome this difficulty, one solution is to add a constant to the transformed undesirable factors to keep them positive [63]. Unfortunately, the determination of this constant has a significant effect on DMU ranking and classification. Färe and Grosskopf [64] propose an alternative approach of using a directional distance function. However, the form of the direction vector could affect the DMU ranking. Thus, in some cases, an improper transformation may generate a result that is inverse from what is expected. According to Guo et al. [65], a widely used form of transformation is reciprocal transformation, which this study adopted based on previous literature [66–69].

To examine the factors that affect the productivity of public hospitals in the Veneto region, we considered institutional factors (i.e., factors that can be controlled by hospital management) and contextual factors (i.e., factors that are beyond the control of hospital management) to estimate their effects on performance. Based on previous literature, we identified five regressors. The first regressor was beds per resident (Beds_Residents), which represents the size of the hospital in relation to the served population [3]. The second regressor was average length of stay (ALOS) [70, 71]. The third regressor was teaching status (Teach), a dummy variable that represents whether a hospital is attached to a university [72]. The fourth regressor was type of hospital [21], another dummy variable that explains whether a structure is labeled a HUB hospital. The fifth regressor was first aid (FIRST-AID) [3], another dummy variable that indicates whether the hospital does or does not provide first aid.

### Model specification

The two basic DEA models are the CCR [40] and the Banker, Charnes, and Cooper (BCC) [73] models. According to Kohl et al. [19], despite countless extensions, almost 80% of studies conducted in the hospital sector apply one of these two basic models. The discussion about which of these two models is superior (and therefore the assumption of constant return to scale [CRS] or variable return to scale [VRS]) has been ongoing since their invention. It is unlikely there will ever be a universally valid answer to that question. However, Banker et al. [74] demonstrate that the CCR model yields better results for small sample sizes of up to 50 DMUs, while the BCC model performs better with larger sample sizes (at least 100 DMUs). Thus, this study used the CCR model. In relation to the choice of model orientation, Alatawi et al. [48] note that the most common orientation employed in studies using DEA analysis are input orientation (i.e., minimizing inputs with a given amount of outputs) and output orientation (i.e., holding inputs constant and proportionally increasing outputs). This study employed input orientation (CCR-I), with the choice being guided by previous literature [43] and because generally, hospitals have more control over their inputs than they do over their outputs. The mathematical formulation is presented below:

$$min\theta -\varepsilon \left(\sum_{i=1}^m{s}_i^{-}+\sum_{r=1}^s{s}_r^{+}\right)$$

subject to:

$$\sum_{j=1}^n{x}_{ij}{\lambda}_j+{s}_i^{-}=\theta {x}_{i0}\ i=1,2,\dots, m;$$

$$\sum_{j=1}^n{y}_{rj}{\lambda}_j+{s}_r^{+}={y}_{r0}\ r=1,2,\dots, s;$$

$${\lambda}_j\ge 0\ j=1,2,\dots, n$$

### Data analysis

A Microsoft Excel database was constructed for all the input, output, and predictor variables using the data provided by Azienda Zero. The Excel data were exported to Gretl software to generate the descriptive statistics. The data were then imported into MaxDEA Basic software to run the CCR-I model, decompose the scores, and assess the slacks.

As stated, when running basic DEA models, it is necessary to choose between CRS or VRS models and the orientation (input or output) of these models. In research, to define scale efficiency (SE), CRS and VRS models are often used simultaneously. The technical efficiency (TE) that can be calculated by CRS models is the overall technical efficiency (OTE). However, VRS specification allows evaluation of PTE to purify the score from the SE effects [73]. From the SE perspective, the sum of lambdas is used to appraise the return to scale (RTS). It is assumed that if a DMU shows a sum of lambdas equal to 1, it operates in CRS; if the sum is less than 1, the DMU has increasing return to scale (IRS); if the sum is greater than 1, the DMU has a decreasing return to scale (DRS). In CRS, the DMU operates in the most productive scale size. In DRS, DMU should reduce its input level, which is referred to as a “downsizing decision.” In IRS, this is opposed to the DRS situation, and the DMU should make an “upsizing decision.”

According to Yildirim [75], it is possible to decompose DEA OTE into its sources of possible inefficiency. This has been achieved using the following formula:

$$\mathrm{OTE}=\mathrm{PTE}\ \mathrm{x}\ \mathrm{SE}$$

CRS (i.e., CCR) and VRS (i.e., BCC) models were established in this study in consideration of the RTS conditions. CRS models assume a constant rate of substitution between inputs and outputs, while VRS models assume that a proportional increase in input level causes a proportionally larger or smaller increase in output level. If CRS and VRS efficiency scores are not equal, this indicates inefficiency of the scale. SE expresses how close the firm is to the optimal scale size: the larger the scale efficiency, the closer the firm is to the optimal scale size [76].

Once the performance of a hospital has been captured by the DEA score, it is useful to attempt to identify some of the factors that could affect efficiency. Kirigia and Asbu [70] identify different regression techniques that have been applied to estimate the effect of contextual factors on efficiency, including ordinary least squares and the maximum-likelihood-based probit, logit, and truncated regression (e.g., Tobit regression). Tobit regression has been widely used in previous literature [3, 70, 77] and was chosen for this study because it can describe the relationship between a nonnegative dependent variable and independent variables. Following Sultan and Crispim [78], the CCR efficiency scores in this study were transformed into inefficiency scores and left-censored at 0 using the following formula:

$$Inefficiency\ score=\left(\frac{1}{CCR\ score}\right)-1$$

Simar and Wilson [79] note that this approach is not without criticism. That is, DEA scores are expected to be reciprocally correlated because the calculation of one DMU includes the observation of all other DMUs and thus suffers from multicollinearity problems. However, according to Banker and Natarajan [80], a DEA approach with Tobit or ordinary least squares functions outperforms the one-stage parametric method (e.g., Cobb–Douglas, Translog) in defining the production frontier.

Accordingly, the estimated Tobit model for the study was specified as follows:

$$\mathrm{INEFFICIENCY}={\upbeta}_0+{\upbeta}_1\mathrm{BedsPop}+{\upbeta}_2\mathrm{ALOS}+{\upbeta}_3\mathrm{Teach}+{\upbeta}_4\mathrm{Type}+{\upbeta}_5\mathrm{FirstAid}+\upvarepsilon,$$

where β is the vector of unknown coefficients and ε is the stochastic/random error term. Using Gretl software, we tested the hypothesis that βn is not significantly different from 0 in either direction. Thus, the null (H0) and alternative hypotheses (HA) are as follows: H0: βn = 0 and HA: βn ≠ 0.

A drawback of basic DEA models is that scores cannot be directly assessed over time. To overcome this issue using DEAP software and following Mujasi and Kirigia [81], we used an input-oriented Malmquist Productivity Index to examine efficiency and productivity changes over the study period (2018–2019). According to Coelli et al. [82], the Malmquist Productivity Index allows identification of the contributions that innovation (technical change) and diffusion and learning (catching up or efficiency change) make to productivity growth. This index attains a value greater than, equal to, or less than 1 if a hospital has experienced productivity growth, stagnation, or productivity decline, respectively. The Malmquist Productivity Index can be broken down into various sources of productivity change, referred to as “technical change” and “efficiency change.” Technical change (TECHCH) is the measure of change in hospital production technology; it measures the shift in technology use over years. Efficiency change (EFFCH) represents change in the gap between observed production and the production frontier between two different years. When estimated under the assumption of CRS, EFFCH can be further broken down into pure efficiency change (PECH) and scale efficiency change (SECH). SECH refers to productivity change resulting from scale change that brings the hospital closer to or further away from the optimal scale of inputs as identified by VRS technology. PECH measures change in TE under the assumption of a VRS technology [81].

## Results

### Descriptive statistics

Descriptive statistics for input and output variables for the years 2018 and 2019 are presented in Table 2.

**Table 2:** Descriptive statistics for input and output variables

**2018**				
	**Mean**	**SD**	**Min**	**Max**
**Beds**	325.16	277.05	42.00	1406.00
**Cost**	107,014,996.79	119,444,746.19	10,448,957.15	617,875,317.22
**FTE Med**	192.46	187.27	18.73	941.73
**FTE Nurse**	619.41	566.30	74.41	3035.33
**FTE Admin**	62.45	95.96	5.00	466.56
**FTE Tech**	130.35	130.11	23.93	638.94
**Outpatient Visits**	1,383,219.35	1,062,499.45	92,484.00	4,145,336.00
**Outpatient Revenue**	21,508,560.21	17,257,859.63	1,625,618.85	72,893,305.05
**Inpatient Revenue**	47,875,980.98	54,576,627.38	3,802,075.90	263,781,278.03
**N. Adj. Inpatients**	13,928.37	15,585.45	516.00	77,165.00
**BOR**	0.74	0.10	0.48	0.98
**Inappropriate Admission**	0.18	0.05	0.11	0.37
**Mortality Rate**	0.04	0.02	0.00	0.11
**2019**				
	Mean	SD	Min	Max
**Beds**	321.37	274.10	41.00	1′419.00
**Cost**	10,670,768.18	119,394,427.57	9,178,451.50	604,145,722.02
**FTE Med**	190.90	187.10	13.29	922.32
**FTE Nurse**	625.85	575.68	73.46	3′069.67
**FTE Admin**	60.07	91.20	3.00	454.88
**FTE Tech**	127.95	126.44	7.44	610.28
**Outpatient Visits**	1,465,891.81	1,157,508.15	119,419.00	4,361,706.00
**Outpatient Revenue**	22,381′605.33	18,750,693.83	1,659,424.85	78,062,444.25
**Inpatient Revenue**	48,210,495.56	55,787,635.72	2,995,648.77	272,635,414.59
**N. Adj. Inpatients**	14,005.53	15,958.86	513.00	79,890.00
**BOR**	0.75	0.10	0.50	0.99
**Inappropriate Admission**	0.16	0.06	0.02	0.34
**Mortality Rate**	0.05	0.02	0.00	0.12

In 2018, the 43 public hospitals under analysis used a total of 13,982 beds, 4,601,644,861.97 of costs, and 43,200.81 of (medical, nursing, administrative, and technical) FTEs to generate 59,478,432 outpatient visits and 2,983,535,271.00 of revenue. In addition, the averages of the numbers of adjusted inpatients and the BOR were 13,928.37 and 74%, respectively, while the inappropriate admission and mortality rate means were 18 and 4%, respectively. In 2019, the number of beds and costs decreased to 13,819.00 and 4,588,391,031.74, respectively, while the number of FTEs in analysis increased to 43,205. units. For output, revenues increased to 3,035,460,338.27 and outpatient visits to 63,033,348 in total. On average, the number of adjusted inpatients, the BOR, and the mortality rate increased to 14,005.53, 75, and 5%, respectively; in addition, the inappropriate admission rate decreased to 16%.

### Overall technical efficiency scores

The results of the first stage of analysis are presented in Table 3.

**Table 3:** Summary of CCR-I scores (2018–19)

2018				2019			
Hospital	Numbers	%	Avg. Efficiency Score	Hospital	Numbers	%	Avg. Efficiency Score
EFFICIENT	28	65.12		EFFICIENT	23	53.49	
INEFFICIENT	15	34.88	0.9301	INEFFICIENT	20	46.51	0.89971
ALL	43		0.9756	ALL	43		0.9533

In 2018, having 28 DMUs were considered efficient, which means that more than 65% of hospitals did not need any input reduction, given the level of quantitative and qualitative outputs. In 2019, 23 DMUs (53%) were considered efficient. The averages of the CCR-I scores were 0.9756 for the first year of analysis (2018), decreasing to 0.9533 in the second year of analysis (2019).

### Pure technical and scale efficiency

The hospitals’ TE, PTE, and SE scores are presented in Table 4.

**Table 4:** OTE, PTE, and SE scores (2018 and 2019)

	2018				2019			
DMU	OTE	PTE	SE	RTS	OTE	PTE	SE	RTS
**H1**	1.0000	1.0000	1.0000	Constant	1.0000	1.0000	1.0000	Constant
**H2**	1.0000	1.0000	1.0000	Constant	0.9794	1.0000	0.9794	Increasing
**H3**	1.0000	1.0000	1.0000	Constant	0.7371	0.8610	0.8561	Increasing
**H4**	0.8884	0.9016	0.9853	Decreasing	0.8745	0.8747	0.9998	Increasing
**H5**	1.0000	1.0000	1.0000	Constant	0.9771	1.0000	0.9771	Decreasing
**H6**	1.0000	1.0000	1.0000	Constant	0.9982	1.0000	0.9982	Decreasing
**H7**	0.9939	1.0000	0.9939	Decreasing	1.0000	1.0000	1.0000	Constant
**H8**	0.9819	1.0000	0.9819	Decreasing	0.9572	0.9573	0.9999	Increasing
**H9**	0.9614	1.0000	0.9614	Decreasing	1.0000	1.0000	1.0000	Constant
**H10**	1.0000	1.0000	1.0000	Constant	1.0000	1.0000	1.0000	Constant
**H11**	1.0000	1.0000	1.0000	Constant	1.0000	1.0000	1.0000	Constant
**H12**	0.7693	0.8105	0.9491	Decreasing	0.7676	0.7947	0.9658	Decreasing
**H13**	0.9978	1.0000	0.9978	Decreasing	1.0000	1.0000	1.0000	Constant
**H14**	0.9871	0.9890	0.9981	Decreasing	0.9318	0.9324	0.9994	Increasing
**H15**	1.0000	1.0000	1.0000	Constant	0.9587	1.0000	0.9587	Decreasing
**H16**	1.0000	1.0000	1.0000	Constant	0.9732	0.9857	0.9873	Decreasing
**H17**	0.9881	0.9882	1.0000	Increasing	1.0000	1.0000	1.0000	Constant
**H18**	1.0000	1.0000	1.0000	Constant	0.7965	0.7993	0.9966	Decreasing
**H19**	0.9198	0.9279	0.9913	Decreasing	1.0000	1.0000	1.0000	Constant
**H20**	0.8660	0.9507	0.9110	Decreasing	1.0000	1.0000	1.0000	Constant
**H21**	1.0000	1.0000	1.0000	Constant	0.8716	0.8806	0.9898	Decreasing
**H22**	1.0000	1.0000	1.0000	Constant	1.0000	1.0000	1.0000	Constant
**H23**	0.9182	0.9295	0.9878	Decreasing	0.8546	0.8557	0.9987	Decreasing
**H24**	1.0000	1.0000	1.0000	Constant	0.8675	0.9103	0.9530	Decreasing
**H25**	1.0000	1.0000	1.0000	Constant	0.8738	0.9517	0.9182	Decreasing
**H26**	0.8260	0.8335	0.9910	Decreasing	1.0000	1.0000	1.0000	Constant
**H27**	1.0000	1.0000	1.0000	Constant	1.0000	1.0000	1.0000	Constant
**H28**	1.0000	1.0000	1.0000	Constant	1.0000	1.0000	1.0000	Constant
**H29**	1.0000	1.0000	1.0000	Constant	1.0000	1.0000	1.0000	Constant
**H30**	0.9315	1.0000	0.9315	Decreasing	0.9186	1.0000	0.9186	Decreasing
**H31**	1.0000	1.0000	1.0000	Constant	1.0000	1.0000	1.0000	Constant
**H32**	1.0000	1.0000	1.0000	Constant	1.0000	1.0000	1.0000	Constant
**H33**	1.0000	1.0000	1.0000	Constant	0.8506	0.8730	0.9744	Increasing
**H34**	1.0000	1.0000	1.0000	Constant	1.0000	1.0000	1.0000	Constant
**H35**	0.9649	0.9763	0.9883	Decreasing	0.8535	0.8660	0.9855	Increasing
**H36**	1.0000	1.0000	1.0000	Constant	0.9699	1.0000	0.9699	Decreasing
**H37**	1.0000	1.0000	1.0000	Constant	1.0000	1.0000	1.0000	Constant
**H38**	1.0000	1.0000	1.0000	Constant	1.0000	1.0000	1.0000	Constant
**H39**	1.0000	1.0000	1.0000	Constant	1.0000	1.0000	1.0000	Constant
**H40**	1.0000	1.0000	1.0000	Constant	1.0000	1.0000	1.0000	Constant
**H41**	0.9572	1.0000	0.9572	Decreasing	0.9827	1.0000	0.9827	Decreasing
**H42**	1.0000	1.0000	1.0000	Constant	1.0000	1.0000	1.0000	Constant
**H43**	1.0000	1.0000	1.0000	Constant	1.0000	1.0000	1.0000	Constant
**Avg**	0.9756	0.9839	0.9913		0.9534	0.9661	0.9863	

As a result of this stage of analysis, overall efficiency was decomposed into pure technical and scale efficiency. It was observed that among the 15 inefficient hospitals in 2018 that were identified, six had only scale inefficiencies, while nine had both managerial and scale inefficiencies. The average TE and SE scores for 2018 were 0.984 and 0.991, respectively. TE scores ranged from 0.810 to 1. For the OTE scores, inefficient hospitals had a minimum score of 0.7692 and a maximum of 0.998, which means that they were able to decrease input usage from 0.2 to 23.08% without changing their qualitative and quantitative output levels. In 2019, 20 hospitals were inefficient. Seven had both inefficiencies (i.e., PTE and SE) and 13 did not have managerial inefficiencies, and only scale inefficiencies. The TE and SE average scores were 0.966 and 0.986, respectively. The minimum TE score was 0.795. Therefore, the results highlight that for both years (2018 and 2019), in most cases, both the input utilization and the scale of the hospitals were causes of hospital inefficiency. The results present an average PTE greater than OTE. In relation to RTS, Table 2 reveals that among the total of 35 inefficient hospitals, 27 presented DRS, which means that 77% of inefficient hospitals needed a downsizing strategy to gain efficiency. The other eight hospitals presented IRS, which indicates that 23% of inefficient hospitals needed to increase their size to gain efficiency. That is, we found a greater prevalence of DRS than IRS in the inefficient hospitals.

### Input and output slack assessment of inefficient hospitals

Inefficiency is caused by noneffective use of inputs and/or outputs [83]. Accordingly, input and output slacks are factors that contribute to inefficiencies. It is important for managers to determine the target values, to measure against targets, and to control their improvement progress. Good operations management decreases output shortage levels (the quantities of output slacks) by employing surplus resources (the quantities of input slacks added to the proportional reduction of inputs [50]). Hence, the evaluation of slack variables is crucial for efficiency improvement. As stated, one of the variables presented (i.e., the number of beds) is not controllable by management because this number is chosen by regional governments in Italy. The input and output slacks of inefficient hospitals are presented in Table 5.

**Table 5:** Input and output slacks of inefficient hospitals

**2018**													
**DMU**	**Slack_Beds** ^a^	**Slack_Cost** ^a^	**Slack_FTE Med** ^a^	**Slack_FTE Nurse** ^a^	**Slack_FTE Admin** ^a^	**Slack_FTE Tech** ^a^	**Slack_Outpatient Visits** ^a^	**Slack_Outpatient Revenue** ^a^	**Slack_Inpatient Revenue** ^a^	**Slack_Adj. Inpatient** ^a^	**Slack_BOR** ^a^	**Slack_Inappropriate** **Admission** ^b^	**Slack_Mortality**_b_
**H4**	0.00%	0.00%	0.00%	−4.24%	−36.66%	−33.57%	24.15%	0.00%	1.02%	0.00%	3.00%	−7.38%	0.00%
**H7**	0.00%	0.00%	0.00%	−2.33%	0.00%	−4.58%	4.60%	0.00%	0.00%	0.00%	12.47%	−2.79%	−2.44%
**H8**	0.00%	0.00%	0.00%	0.00%	0.00%	0.00%	54.65%	22.82%	5.01%	0.00%	10.05%	−4.64%	−1.49%
**H9**	0.00%	0.00%	0.00%	0.00%	−14.05%	0.00%	0.00%	0.00%	0.00%	0.00%	15.47%	−5.47%	− 3.43%
**H12**	0.00%	−7.23%	0.00%	−13.68%	0.00%	0.00%	0.00%	0.00%	0.00%	0.31%	7.63%	−4.32%	−3.44%
**H13**	0.00%	0.00%	−4.23%	−7.80%	0.00%	−4.61%	0.00%	0.00%	0.55%	0.00%	17.13%	−2.09%	−5.56%
**H14**	0.00%	0.00%	−15.11%	−15.88%	−40.13%	0.00%	64.61%	16.15%	0.00%	0.00%	0.00%	0.00%	−0.97%
**H17**	0.00%	0.00%	−5.16%	0.00%	0.00%	−19.31%	0.00%	0.00%	0.00%	1.01%	0.34%	0.00%	−2.94%
**H19**	0.00%	−4.71%	− 13.65%	0.00%	0.00%	−22.91%	21.69%	0.00%	5.48%	0.00%	0.00%	−3.77%	−4.63%
**H20**	0.00%	0.00%	0.00%	−8.84%	−0.49%	−7.32%	30.03%	0.00%	0.00%	2.27%	11.12%	−4.12%	−7.73%
**H23**	0.00%	0.00%	0.00%	0.00%	−41.82%	− 8.91%	173.03%	0.00%	0.00%	4.14%	12.38%	−3.07%	−1.22%
**H26**	0.00%	0.00%	0.00%	−0.02%	−25.75%	−6.66%	0.00%	0.00%	3.97%	0.00%	7.38%	−6.09%	−4.61%
**H30**	0.00%	0.00%	0.00%	0.00%	−7.25%	−4.03%	0.00%	0.00%	0.00%	7.13%	15.55%	−2.78%	−4.01%
**H35**	0.00%	0.00%	−4.26%	−16.81%	−44.99%	0.00%	21.89%	60.61%	4.52%	0.00%	0.00%	−13.65%	−1.06%
**H41**	0.00%	−8.37%	0.00%	−6.70%	0.00%	−0.01%	16.50%	32.08%	2.82%	0.00%	12.35%	−1.83%	−0.60%
**AVG 2018**	0.00%	−1.35%	−2.83%	−5.09%	−14.08%	−7.46%	27.41%	8.78%	1.56%	0.99%	8.32%	−4.13%	−2.94%
**2019**													
**DMU**	**Slack_Beds** ^a^	**Slack_Cost** ^a^	**Slack_FTE Med**_a_	**Slack_FTE Nurse**_a_	**Slack_FTE Admin** ^a^	**Slack_ FTE Tech** ^a^	**Slack_** **Outpatient Visits** ^a^	**Slack_** **Outpatient Revenue** ^a^	**Slack_** **Inpatient Revenue** ^a^	**Slack_** **Adj. Inpatient**_a_	**Slack_BOR**_a_	**Slack_** **Inappropriate** **Admission**_b_	**Slack_ Mortality** ^b^
**H2**	−10.34%	0.00%	−36.65%	−2.92%	−62.26%	− 32.90%	30.28%	3.28%	8.39%	0.00%	0.00%	−9.16%	−4.40%
**H3**	0.00%	−12.36%	0.00%	−14.29%	0.00%	−27.06%	0.00%	0.00%	76.81%	19.44%	0.00%	0.00%	−10.69%
**H4**	0.00%	0.00%	−7.09%	0.00%	−28.11%	−25.07%	0.00%	0.00%	0.00%	0.00%	0.00%	−4.70%	−0.08%
**H5**	−0.63%	−4.33%	0.00%	0.00%	−10.86%	0.00%	0.00%	0.00%	0.00%	2.73%	14.05%	−2.12%	−3.00%
**H6**	−26.96%	−18.54%	−20.04%	−18.45%	−78.52%	0.00%	36.71%	0.00%	0.00%	3.91%	27.90%	−8.73%	0.00%
**H8**	−16.81%	0.00%	−24.40%	0.00%	−28.53%	−14.86%	0.00%	0.00%	5.94%	0.00%	0.00%	−3.34%	−3.26%
**H12**	0.00%	−7.09%	0.00%	−3.61%	0.00%	0.00%	0.00%	0.00%	0.00%	5.01%	0.00%	−4.12%	−4.19%
**H14**	0.00%	0.00%	−19.39%	−15.44%	−13.80%	0.00%	32.38%	12.75%	0.00%	2.66%	0.00%	−4.04%	−1.65%
**H15**	0.00%	−5.15%	−3.47%	−15.74%	−30.15%	0.00%	0.00%	0.00%	0.00%	4.81%	4.33%	−15.21%	−5.46%
**H16**	−6.51%	0.00%	−8.80%	0.00%	−7.19%	−22.30%	0.00%	0.00%	0.00%	4.31%	0.00%	−4.87%	−1.84%
**H18**	−3.72%	0.00%	−2.79%	0.00%	−3.56%	−34.72%	0.00%	0.00%	1.54%	0.00%	0.00%	0.00%	−10.54%
**H21**	−2.30%	0.00%	−15.26%	0.00%	0.00%	−2.39%	0.00%	0.00%	0.00%	2.64%	16.98%	−10.34%	−6.10%
**H23**	0.00%	0.00%	−14.44%	0.00%	−22.23%	−5.99%	84.72%	0.00%	0.00%	7.17%	6.39%	−3.07%	−2.84%
**H24**	0.00%	0.00%	−8.28%	0.00%	−9.74%	0.00%	0.00%	0.00%	0.00%	1.02%	0.00%	−4.86%	−6.05%
**H25**	0.00%	0.00%	−19.32%	−6.35%	0.00%	0.00%	0.00%	0.00%	0.00%	1.44%	8.60%	−6.59%	−5.77%
**H30**	0.00%	0.00%	−0.24%	−3.81%	−36.97%	−12.26%	0.00%	0.00%	0.00%	4.85%	5.30%	−8.70%	−2.24%
**H33**	−16.83%	0.00%	−26.71%	−11.20%	−45.18%	−30.41%	16.71%	78.74%	0.00%	19.10%	0.00%	−13.96%	−2.11%
**H35**	0.00%	0.00%	−33.02%	0.00%	−71.35%	−14.18%	14.14%	32.83%	3.51%	0.00%	0.00%	−3.54%	−1.04%
**H36**	0.00%	0.00%	−10.86%	0.00%	−38.17%	0.00%	0.00%	0.00%	0.00%	3.12%	14.27%	−4.27%	−5.13%
**H41**	0.00%	−4.77%	0.00%	−7.56%	0.00%	0.00%	21.55%	38.09%	2.28%	0	12.75%	−1.74%	−1.34%
**AVG 2019**	−4.21%	−2.61%	−12.54%	−4.97%	−24.33%	− 11.11%	11.82%	8.28%	4.92%	4.11%	5.53%	−5.67%	−3.89%
**AVG**	−2.40%	−2.07%	−8.38%	−5.02%	−19.94%	−9.54%	18.50%	8.50%	3.48%	2.77%	6.73%	−5.01%	−3.48%

^a^ The results have been transformed into percentages

^b^ To ensure a better reading of the results, the undesirable outputs results have been retransformed into percentages by reciprocal transformation

In this table, the minus sign indicates that a further reduction is necessary to obtain an improvement for the variable; the plus sign indicates that a further increment of the variable is required. The results show that beyond the proportional movement, the inputs that were controllable by management and that needed a particularly significant reduction were, on average, FTE administrative staff and technicians. The output that required the biggest increase was the number of outpatient visits. The analysis reveals that a significant reduction of the quality output variables was not needed.

### Internal and external sources of inefficiency

The selected Tobit model for explaining the observed hospital inefficiencies in stage four of the analysis was presented in the Data Analysis section. Table 6 presents the results of the Tobit regression model.

**Table 6:** Results of Tobit model

Variable	Coefficient	t	***p***-value
**Beds_Residents**	51.60629	2.39358	0.01668*
**ALOS**	0.00897	2.01891	0.04349*
**Type**	−0.13280	−1.71839	0.08572
**Teach**	−0.00771	−0.08168	0.93489
**FIRST-AID**	−0.07154	−1.17805	0.23877
**Const**	−0.12795	−1.69440	0.09019

* *p*-value < 0.05

The results reveal that the coefficient for Beds_Residents (i.e., the size per population served) had a positive sign, statistically significant at the 5% level. This means that the higher a hospital’s Beds_Residents, the higher the predicted inefficiency score. The relationship between size and efficiency remains controversial in the literature. In this study, Tobit regression analysis indicated that size negatively affected the efficiency scores. Average length of stay (ALOS) was the second significant variable (*p* < 0.05) and had a positive sign, which means that it was positively correlated with inefficiency. The other three regressors (i.e., type of hospital, teaching status, and first-aid provision) were not statistically significant at the 5% level.

### Efficiency evaluation over time

Table 7 presents the Malmquist Productivity Index summary of annual geometric means.

**Table 7:** Malmquist Productivity Index results

	EFFCH (A = CxD)	TECHCH (B)	PECH (C)	SECH (D = A/C)	TFPCH (E = AxB)
**H1**	1	1.069	1	1	1.069
**H2**	1	1.148	1	1	1.148
**H3**	1	1.239	1	1	1.239
**H4**	1.012	0.88	1.005	1.007	0.89
**H5**	1	1.119	1	1	1.119
**H6**	1	1.573	1	1	1.573
**H7**	0.886	0.646	1	0.886	0.572
**H8**	1	0.816	1	1	0.816
**H9**	0.904	0.94	0.921	0.982	0.85
**H10**	1.449	1.027	1.222	1.186	1.488
**H11**	0.988	0.435	1	0.988	0.43
**H12**	1.082	1.08	1	1.082	1.169
**H13**	1	1.626	1	1	1.626
**H14**	0.565	0.784	0.764	0.74	0.443
**H15**	0.899	1.118	1.047	0.859	1.005
**H16**	1.532	0.966	1.018	1.505	1.481
**H17**	0.893	1.159	0.915	0.976	1.035
**H18**	1	0.737	1	1	0.737
**H19**	0.894	1.013	0.979	0.913	0.906
**H20**	1.823	1.326	1	1.823	2.417
**H21**	0.752	0.995	1.046	0.719	0.748
**H22**	1	1.236	1	1	1.236
**H23**	1.034	0.836	0.991	1.044	0.865
**H24**	0.847	0.693	1	0.847	0.587
**H25**	1	0.528	1	1	0.528
**H26**	1	0.975	1	1	0.975
**H27**	0.745	0.285	0.918	0.812	0.212
**H28**	1	0.817	1	1	0.817
**H29**	0.569	0.492	0.844	0.674	0.28
**H30**	1	0.959	1	1	0.959
**H31**	0.774	1.122	1	0.774	0.868
**H32**	1	0.706	1	1	0.706
**H33**	1	1.285	1	1	1.285
**H34**	1	0.875	1	1	0.875
**H35**	1	0.718	1	1	0.718
**H36**	0.912	1.086	1	0.912	0.991
**H37**	1	0.976	1	1	0.976
**H38**	1.046	1.269	1.037	1.008	1.327
**H39**	1.111	1.943	1.111	1	2.16
**H40**	1	1.186	1	1	1.186
**H41**	1	0.125	1	1	0.125
**H42**	1	1.104	1	1	1.104
**H43**	1	1.627	1	1	1.627
**Mean**	0.974	0.908	0.994	0.98	0.885

Note: All Malmquist Productivity Index averages are geometric means

Analyzing the total factor productivity change (TFPCH) (i.e., the Malmquist Productivity Index) in the last column number 6 of Table 7 reveals that on average, TFPCH decreased slightly by 11.5% over the 2018–2019 period. The minimum score was 0.125 and the maximum score was 2.417. Nineteen (44.2%) hospitals had TFPCH scores greater than 1, indicating growth in productivity. The productivity growth in 13 hospitals was attributed to technological innovation only (with EFFCH equal to or less than 1); one hospital performed better because of efficiency improvements only. In contrast, 24 hospitals (55.8%) had a TFPCH of less than 1, indicating a decrease in productivity. Productivity decrease in three hospitals was because of a decline in efficiency only. In nine hospitals, productivity decrease was attributed to a decrease in both efficiency and innovation, while in 12 hospitals, productivity decrease was attributed to a decrease in technological innovation. PECH, which refers to management efficiency change, and SECH, which refers to scale efficiency change, are the two components of EFFCH. PECH had an average score of 0.994 and SECH had an average score of 0.98. Seven hospitals had a PECH greater than 1, 29 equal to 1, and seven less than 1. The SECH scores indicate that seven hospitals increased their scale efficiency, 23 hospitals kept their scale efficiency constant, and 13 decreased their scale efficiency. The PECH and SECH results reveal that the major cause of the decrease was TECHCH (0.908) rather than EFFCH (0.974).

## Discussion

Analyzing hospital efficiency is a highly important area of research in healthcare management [84] because it enables identification of inefficiency sources and supports corrective action so that scarce resources are not wasted. Using an input-oriented CCR model in which even the quality variables (i.e., mortality rate and inappropriate admission rate) were held constant, the analysis reveals that on average, the efficiency of hospitals in the Veneto region was high and was even higher than has been found in previous literature [3].

For most of the inefficient hospitals identified in the study, the results highlight that for the two years under investigation, both input utilization and hospital scale caused inefficiency. Previous literature reports an average PTE that is greater than the OTE [75, 84].

In relation to the inefficient hospitals, the study highlights the importance of making improvements in both policymaking and managerial capacities. According to the scale efficiency analysis, hospital size (i.e., the number of beds) is one of the most important features in inefficiency. That is, in relation to RTS, the results are consistent with those of Kirigia and Asbu [70] and Tlotlego [85], showing that there was a prevalence of DRS over IRS for inefficient hospitals, meaning that most inefficient hospitals needed a downsizing strategy to gain efficiency.

In addition, the findings of the slack assessment suggest that the inefficient hospitals needed to reorganize FTEs, particularly improving the efficiency of administrative and technical staff.

The results for the analysis of the external and internal factors related to efficiency show partial agreement with previous studies. Specifically, in line with Rebba and Rizzi [24] and Nayar et al. [27], the Tobit regression analysis in our study indicates that hospital size negatively affects efficiency scores. However, these results differ from the findings of Daidone and D’Amico [25]. Average length of stay (ALOS) was also a significant variable (*p* < 0.05). It had a positive sign, which means that it was positively related to inefficiency, and this correlation has been identified in several other studies [22, 23].

Unlike Kakeman et al. [21], we found that the type of hospital was a nonsignificant driver of efficiency. We also found that teaching status was a nonsignificant factor of efficiency, which aligns with the findings of Kakeman et al. [21] but contrasts with those of Sarabi Asiabar et al. [86] and Ali et al. [87]. In addition, the nonsignificance of the finding for the presence of first-aid provision differs greatly from previous literature [28, 29].

In assessing efficiency over time, we found that the major cause of the decrease in productivity was technical change rather than efficiency change. The finding that average technical change was lower than average efficiency change is in line with the findings of Li et al. [88] and Tlotlego et al. [85]. Technological progress (or decline) depends on different factors, including the availability of appropriate healthcare technology, access to new technologies at affordable prices, the availability of training opportunities that enable the workforce to acquire new skills and take full advantage of new technological possibilities, and the availability of funds to finance necessary healthcare technology investments [89].

### Limitations and further research

This study has some limitations. Beyond the typical limitations of basic DEA models [19], some issues are specific to this study. One is that we incorporated only a limited number of quality measures into the model, excluding measures such as hospital readmission rates because of the unavailability of data. In addition, an examination of demographic factors such as the age of population served could reveal significant information for the hospitals we analyzed. In addition, further research could extend this study by assessing hospital efficiency in other Italian regions.

## Conclusions

This study contributes to the healthcare management literature because few previous studies in the European context have analyzed hospital efficiency using quality measures as outputs [36]. To fill this research gap, this research used hospitals in the Veneto region as a case study, identifying the organizational causes that affected efficiency, also considering quality measures, and how these changed over a period of two years. The results reveal that more than half of the hospitals under review were efficient. For the hospitals that were found to be inefficient, many had both input utilization and scale inefficiency. This study also provides empirical evidence for the main causes of inefficiency. Hospital size is one of the most important sources of inefficiency. In addition, administrative and technical staff often cause inefficiency. The contextual factors that most influence efficiency are the average patient length of stay and hospital size with respect to the population served. The role of technology is crucial to maintaining or increasing efficiency levels over time.

DEA is a good method for measuring hospital performance, in addition to the budgeting process that has traditionally been used in Italy. DEA is particularly useful for its ability to estimate the volume of inputs and outputs that can be optimized and its capacity to identify the main sources of inefficiency.

The results also underscore that a rethink of hospital size on the part of policymakers would be particularly valuable for increasing the current efficiency levels of many hospitals and maintaining constant and high service quality. Improving performance also depends on improving staff efficiency, particularly administrative and technical staff. This may be achieved by providing capacity building such as training staff members on efficient resource utilization. Finally, the results suggest that hospital managers should pay significant attention to advancements in technology and the skills needed to employ new technology in the best possible way.

## Data Availability

The data that support the findings of this study are available from Azienda Zero—Veneto Region, but restrictions apply to the availability of these data, which were used under license for the current study, and so are not publicly available. However, data are available from the authors upon reasonable request and with the permission of Azienda Zero—Veneto Region.

## Abbreviations

ALOS: Average length of stay
BCC: Banker, Charnes, and Cooper
BOR: Bed occupancy rate
CCR: Charnes, Cooper, and Rhodes
CRS: Constant return to scale
DEA: Data envelopment analysis
DMU: Decision-making unit
DRS: Decreasing return to scale
EFFCH: Efficiency change
FTE: Full-time employee
IRS: Increasing return to scale
OTE: Overall technical efficiency
PECH: Pure efficiency change
PTE: Pure technical efficiency
RTS: Return to scale
SE: Scale efficiency
SECH: Scale efficiency change
TE: Technical efficiency
TECHCH: Technical change
TFPCH: Total factor productivity change
VRS: Variable return to scale.
